# Hay Asthma

**Published:** 1853-11

**Authors:** 


### *Hay Asthma.* By Wm. C. Wey, M. D., Elmira, N. Y.

In June, 1852, I was consulted by a lady respecting a variety of disease, of which I had frequently read entertaining descriptions in books, but had never before been permitted to witness. I refer to what is commonly known as "hay asthma."

Nine years previously, at a period when innumerable flowers and grasses were appearing, and the air was laden with their aroma, the subject of this record was attacked with what was considered common catarrh, the intensity of which passed away, leaving the mucous membrane of the air-passages peculiarly susceptible to impressions of offending odors. During the summer of 1843, Mrs. B. suffered many repetitions of the catarrhal disease, which entirely disappeared after the first severe autumnal frost. Again, in the spring of 1844, on the expansion of vegetation, she experienced a return of the disorder, without for a moment imagining its re-appearance at that time as anything more than accidental, or ascribable to the changeable season and increased out-of-door employment. She was soon, however, taught by experience, that her sufferings were at all times increased by inhaling the odor of flowers, and found it decidedly painful and distressing to breathe the atmosphere of gardens and meadows. The advice of a physician in New Hampshire, where our patient resided, was sought, and all doubt concerning the nature and cause of her sickness was dispelled by his advice and information on the subject.

The following narration of the symptoms assumed in this interesting case, Mrs. B. has manifested during the spring and summer for nine successive years, and esteems her lot hard indeed, to be forbidden the pleasure of indulging her natural taste in the cultivation and arrangement of ornamental flowers and shrubs.

Early in June, frequently in May, as soon as roses and other highly odorous flowers appear, she is attacked with suffusion of the eyes, constant sneezing, accompanied by the escape of thin mucus from the nostrils—suffers exquisite pain, like neuralgia, in the orbits and brow—experiences a sensation of constriction in the chest, as if a band was tightly drawn around the waist—breathes hurriedly and anxiously—has a dry, irritating cough, and for a longer or shorter time, depending on the continuance of the offending cause, is prostrated, confined to her bed, and wholly incapacitated from attending to her ordinary, or even commonest duties; in an hour or two, generally, there is an abatement of all the symptoms, the breathing becomes quiet and regular, the cough subsides, the feeling of suffocation is removed, and she experiences relief similar to that which follows or concludes an attack of spasmodic asthma, when the cough softens down, with an abundant and easily removed expectoration.

Paroxysms of this description were suffered in the morning, at noon, and in the evening, with great regularity, and with the single exception of the cough, a complete intermission occurred between them, and her mental and physical powers resumed again their wonted activity and vigor.

This disease or infliction is not confined to the summer months, but is developed in mid-winter and at other seasons, by inhaling the aroma of flowers. It so readily follows the least escape of floral perfume, that she cannot remain in an apartment in which flowers are preserved, as I had an opportunity of witnessing during an interview at my office. Although her evening paroxysm had subsided, and by avoiding exposure to the exciting cause of the dyspnœa, she hoped to escape further inconvenience during the remainder of the day; yet, after sitting a few minutes, I was compelled to remove from a table two or three rose-buds, which I had preserved in water, on account of the sudden development of an incessant, dry, hacking cough, similar to what she had often experienced on walking or riding through a garden.

The peculiarities of this case differ in no essential respects from the instances of the affection mentioned in works of practical medicine, and my purpose in presenting it to the Association, is to bear testimony to the efficiency of the remedy with which I succeeded in averting its disagreeable course.

The recurrence of the disease, year after year, and its absolute dependence on the inhalation of gases developed by grasses and flowers, and its occasional production in the winter months, by exposure to the same irritating cause, suggested the propriety of removal from the country to the sea-shore, during the summer and early autumn months. A few years before, Mrs. B. availed herself of this promise of relief, and spent several weeks at the sea-side and on the water; but instead of yielding abatement to her sufferings, the sea air or some other agency considerably aggravated the disorder.

Considering this, it became necessary to prescribe for the relief of an affection developed and continued by a well-known though subtle cause, whose operations offered the strongest impediment to the successful employment of remedial agents. I advised avoidance, as completely as could be done, of localities highly charged with the odor of flowers and grasses, and gave hydrocyanic acid, in mucilage, to soothe and subdue the cough, which was the most prominent and troublesome symptom. Taking into consideration the *periodical* tendency of the disease, I did not hesitate to recommend the liberal employment of quinine, or some preparation of arsenic, should the prussic acid prove unavailing. The prescription was as follows:

℞ Mucilage, ℥ij,
Hydrocyanic acid, m. xxx.
Fifteen drops every two hours. M.

Almost immediately after commencing its use, the cough yielded, and on the following day, no paroxysm of the dyspnœa returning, she felt greatly encouraged to continue the remedy. During the remainder of the summer, part of which she spent in Elmira, and part in New Hampshire, she experienced only the slightest inconvenience from the disease, though on many occasions she rode several miles in the country, and was frequently and variously exposed to the peculiar influences which, but a short time before, never failed to develop an attack. She was never without the remedy for a day, until the cold weather set in, and used it with extreme faithfulness and confidence.

Hay fever, hay asthma, summer catarrh, and "sea-cold," as my patient designated her affection, or idiosyncrasy, is considered by Dr. Elliotson as a combination of catarrh and asthma, and by Dr. Dunglison is called a "singular variety of chronic bronchitis." It is oftentimes an hereditary disease, successively appearing in parent and child, and of all maladies, is, in particular instances, the most intractable. It is aggravated by the use of fruits, by exposure, not only to the offending and specific cause, but to extremes of temperature, by excitement of mind, &c.

A case is related in Dunglison's Practice, on the authority of Mr. Poyan, in which all the symptoms of the disease were produced by the smell of a guinea-pig; and I once knew a gentleman who could not remain in an apartment in which apples were preserved, without experiencing dyspnœa, headache, constriction of the chest, and other unpleasant symptoms; and another instance of an individual to whom cheese was peculiarly obnoxious, on account of the production of the same phenomena.

Respecting the treatment of hay asthma, much has been written, and various methods have been proposed. Previous to an anticipated attack, the cold shower-bath has been found especially serviceable as a prophylactic, followed by quinine and sulphate of iron, according to the plan propounded by Mr. Gordon, an English writer. In speaking of this combination, he states that it proved "eminently successful in emancipating from this tormenting disorder, two patients, whose cases he had previously related; who, in spite of all other treatment, suffered an annual return of it for fifteen years."

Chloride of lime was recommended by Dr. Elliotson, to a sufferer, on the principle of its efficiency in destroying *animal* effluvia, and liberal use was made of it in the sleeping-room and other apartments of the house, with complete success. "Three patients out of five derived advantage from it." Dr. Watson suggests a trial of the *respirator*, as a defense against particles of ipecacuanha, and against the volatile exciting cause (whatever it may be) of hay asthma.—*Trans. Med. Association Southern Central New York.*

